# Brainwashing the cybernetic spectator: *The Ipcress File*, 1960s cinematic spectacle and the sciences of mind

**DOI:** 10.1177/0952695117703295

**Published:** 2017-07-13

**Authors:** Marcia Holmes

**Affiliations:** Birkbeck, University of London, UK

**Keywords:** brainwashing, cybernetics, history of film, neuroscience, spectatorship

## Abstract

This article argues that the mid-1960s saw a dramatic shift in how ‘brainwashing’ was popularly imagined, reflecting Anglo-American developments in the sciences of mind as well as shifts in mass media culture. The 1965 British film *The Ipcress File* (dir. Sidney J. Furie, starr. Michael Caine) provides a rich case for exploring these interconnections between mind control, mind science and media, as it exemplifies the era’s innovations for depicting ‘brainwashing’ on screen: the film’s protagonist is subjected to flashing lights and electronic music, pulsating to the ‘rhythm of brainwaves’. This article describes the making of *The Ipcress File*’s brainwashing sequence and shows how its quest for cinematic spectacle drew on developments in cybernetic science, multimedia design and modernist architecture (developments that were also influencing the 1960s psychedelic counter-culture). I argue that often interposed between the disparate endeavours of 1960s mind control, psychological science and media was a vision of the human mind as a ‘cybernetic spectator’: a subject who scrutinizes how media and other demands on her sensory perception can affect consciousness, and seeks to consciously participate in this mental conditioning and guide its effects.

In the early years of the cold war, Americans and their allies were frequently invited to imagine ‘brainwashing’ as a new and dangerous threat to democratic civilization. Stories about brainwashing circulated widely in the United States and beyond as western citizens tried to make sense of new enemies and new forms of warfare that emerged after the Second World War (Carruthers, 2009; Melley, 2012; Seed, 2004). Befitting the postwar era’s many innovations in visual media, ‘brainwashing’ gained an iconography, apparent in films and television shows, as well as a body of literature. Tracking the history of changing popular beliefs about brainwashing is an admittedly complex undertaking, but the visual imagery surrounding brainwashing seems to have undergone a straightforward transformation. At first, in the 1950s, representations of brainwashing were inspired by real events that took place in countries ruled by communist parties. By the mid-1960s, more fantastic visions of brainwashing became common, and often brainwashing appeared as futuristic, overtly scientific processes that were as incredible as they were entertaining. As the cold war wore on, film and television increasingly did more than portray the era’s evolving imagery of brainwashing: they served as models for how Americans and their allies imagined the possibilities of mind control. In such films as *The Manchurian Candidate* (1962), *A Clockwork Orange* (1971), *The Parallax View* (1974) and *Videodrome* (1980), the historian Andreas Killen observes, visual media are themselves depicted as precision tools for conditioning and coercing audiences (Killen, 2011).

Within the history of ‘brainwashing’, as with the histories of hypnosis and mesmerism, it is possible to trace the interweaving of at least three threads: the popular imagery of mind control; scientific discourses on what makes a human mind vulnerable to influence; and the era’s favoured forms of mass media for communicating these images and ideas. Indeed, these entanglements were not just fundamental to the cold war imaginary surrounding brainwashing, but a continuation of the long history of fearing the dangerous allure of popular entertainment. Much earlier in the century, similar anxieties had seemed to link hypnosis, cinema and psychoanalytic interpretations of the unconscious (Andriopoulos, 2008; Bergstrom, 1979). Certainly, post-1945 debates about brainwashing at times echo the *fin-de-siècle*’s pervasive fascination with suggestibility and what Jonathan Crary has called ‘spectacular culture’ (Crary, 2001). Yet, alongside these continuities, the 1950s and 1960s saw significant shifts in the popular imagery of mind control, the means of mass communication, and the ideas and practices of the mind sciences. These developments could at times cohere, I suggest, around an emerging, postwar vision of the human subject, a model for understanding media spectatorship, which interpreted the mind’s engagement with the sensory delights of media using terms and concepts taken from postwar cybernetic philosophy. Such a vision held that the mind was inherently vulnerable to sensory perception, to conditioning from the environment; and yet, these mental processes could be consciously intervened in, even guided, by the spectator as a means to resist coercion or gain enlightenment. As historians, we might call this model of mind the ‘cybernetic spectator’, and note the ways that it appears in discussions about mind control and media during the 1960s and beyond.

This article shows how the evolving imagery of brainwashing, especially as it seems to turn away from psychological realism to futuristic fantasy, intersects with this popular-cybernetic interpretation of mind and also, relatedly, the 1960s practices of spectacular media. I offer the 1965 British film *The Ipcress File*, directed by Sidney J. Furie and starring Michael Caine, as a useful case for exploring the relationship between popular brainwashing imagery and transatlantic developments in science, modern art and multimedia communication. Recent scholarship on the cultural imagery surrounding brainwashing has tended to focus on *The Manchurian Candidate*, the 1962 film directed by John Frankenheimer (Andriopoulos, 2011; Carruthers, 1998; Jacobson and Gonzalez, 2006; Killen, 2011; Melley, 2011; Seed, 2004; Winter, 2011). Though it has received far less attention, *The Ipcress File* illuminates a particular vision of ‘brainwashing’ that was imagined, seemingly for the first time, in the mid-1960s: a consciously cinematic, almost psychedelic, composition of flashing images and pulsating noises that, the film suggested, might reprogramme a spectator’s consciousness.

The interactions between the mind sciences, mind control and the apparatus of audio-visual media have previously been explored by historians such as Stefan Andriopoulos, Andreas Killen and Alison Winter (Andriopoulos, 2008, 2011; Killen, 2011, 2012; Killen and Andriopoulos, 2011; Winter, 2004, 2006, 2012). My argument raises for discussion how postwar visions of the human mind, such as those informed by cybernetics and neuroscience, could, in the 1960s, guide and reflect the constantly evolving relationship between the mind sciences, mass media and popular fantasies about influence. This analysis of *The Ipcress File*, moreover, helps to extend the history of brainwashing, as a cultural and scientific concept, beyond the American public sphere to broader transatlantic audiences and discourses. Though the concept of brainwashing, as we generally comprehend it, was certainly an American invention, the human sciences and forms of media that moulded brainwashing’s meanings over time were transnational and responded to innovations on both sides of the Atlantic.

Below, I give a short history of films and television series about brainwashing, and suggest that their fantastical imagery was encouraged by structural changes in the production of visual media and a trend known as ‘spy mania’. I argue that *The Ipcress File* exemplifies this trend’s turn toward transatlantic audiences and consciously spectacular imagery. I then elaborate *The Ipcress File*’s depiction of brainwashing, explaining the sources of inspiration for its script and iconic set, in order to show how the film drew on contemporary innovations in neuroscience and modernist design, developments that would also influence the experimental art of 1960s counter-cultures. The serendipitous overlap between *The Ipcress File* and psychedelic art, as the final section of this article will discuss, indicates a deeper transformation in how artistic spectacle was created, communicated and experienced in the 1960s. Arguably, this transformation cast the human mind as a ‘cybernetic spectator’: a subject who not only scrutinizes how media and other demands on his or her sensory perception can affect consciousness, but seeks to consciously participate in this mental conditioning and guide its effects.

## Brainwashing on film

The term ‘brainwashing’ was introduced into English in 1950, though its various meanings and referents have much longer, deeper histories (Carruthers, 2009; Dunne, 2013; Gleason, 1995). That year, the American journalist Edward Hunter presented the term to his anglophone readers as a translation of the Chinese phrase ‘*hsi nao’* [to wash the brain], saying that it was coined by the Chinese to describe the techniques then being used by Chinese communists to forcibly indoctrinate adults and children in Maoist ideology (Hunter, 1950, 1951). The idea of ‘brainwashing’ quickly came to encompass other nefarious acts by communists, including the interrogation practices of the secret police in the Soviet Union and other communist-run states, and the treatment of American soldiers and their Allies who were captured by communist forces in the Korean War (Lemov, 2015; Streatfeild, 2007; Young, 2014). By the end of the 1950s, ‘brainwashing’ had become shorthand for all manner of anxieties about the vulnerabilities of individuals to external influence – a proliferation that was encouraged by sensationalist journalism, popular works of fiction and even sober, scientific accounts.

Indeed, the era’s expert accounts of actual communist techniques for indoctrination and interrogation were, by their authors’ own admission, barely effective in guiding popular concepts of brainwashing. In 1962 the social psychologist Albert Biderman took stock of Americans’ changing, sometimes convoluted beliefs about brainwashing to explain why, despite recent scientific reports on actual communist practices, popular fantasies about mind control dominated public debate. Biderman noted how, in its original formulation, ‘brainwashing’ was something that happened within communist regimes that were distant, geographically as well as ideologically, from western democracies. This was true both for techniques of mass indoctrination believed to be used by the Chinese, and for coercive interrogation practices of the Soviet secret police – the two phenomena most closely associated with ‘brainwashing’ in this early phase. The fundamental ‘otherness’ of brainwashing meant, as Biderman explained, that ‘Unlike many matters with which social scientists deal, “brainwashing” was not an issue regarding which every journalist, much less everyman, felt he was his own expert’ (Biderman, 1962: 551).

How westerners thought about brainwashing evolved during the Korean War, Biderman believed, when American servicemen were captured by communist forces and held in prisoner-of-war camps until the end of the conflict in 1953. The US Army and the US Air Force, concerned that these men might have been brainwashed, commissioned psychiatrists and psychologists like Biderman to study POWs as they returned to the United States. These behavioural scientists were tasked with determining what had happened in the communist camps and whether the repatriated POWs would be a threat to American society. As the first experts to embark on systematic, empirical studies of brainwashing, Biderman and his colleagues argued that real communist methods were neither mystical nor scientifically advanced, but instead were well-known techniques of interrogation such as solitary confinement, sleep deprivation, hunger and standing still for long periods of time. POWs had also been forced to listen to communist propaganda, and participate in group discussions where conciliatory, collaborative behaviour was rewarded and resistance was punished. Behavioural scientists made an effort to debunk popular claims that the POWs were subjected to a special form of ‘Pavlovian’ conditioning, hypnosis or drugs (1962: 550).^1^ These revelations had some effect on serious public discussion, Biderman claimed, as ‘explanations by Western writers changed from a concern with how strange people responded to a strange technique…to how not-so-strange people responded to not-so-strange techniques’ (ibid.: 556). However, Biderman bemoaned, the genie was already out of the bottle. The emerging facts about brainwashing phenomena did not stop Americans and their allies from fantasizing wildly about what might have happened to the victims of communist coercion.

Subsequent historical accounts have justified Biderman’s bemused frustration with Americans’ willingness to entertain fanciful notions. Indeed, in the same year that Biderman’s essay appeared, the John Frankenheimer film *The Manchurian Candidate* (1962) opened in cinemas. *The Manchurian Candidate* blatantly bypassed scientific opinion in its story of a returning POW who has been brainwashed to serve as an assassin. *The Manchurian Candidate* can be seen as a turning point in the history of brainwashing imagery on screen, not least for how it so wilfully presented, perhaps even parodied, Americans’ fears about what had happened to POWs in Korea (Carruthers, 1998). Yet, this turn was not simply a rejection of scientific expertise, as Biderman’s account might imply, but from one mode of popular spectacle to another: from sensational stories about the ‘truth’ of what happens to communist captives to deliberately incredible fictions about the covert forces that imperil western citizens. While mind control had long been a fixture of B-movies, in the 1950s the mainstream films that addressed communist brainwashing often took a literal approach. Films like *Guilty of Treason* (1950), *Assignment-Paris!* (1952), *Prisoner of War* (1954), *The Rack* (1956) and a British production starring Alec Guinness, *The Prisoner* (1955), all claimed to reveal the ‘real’ story, or at least a plausible one, of what had happened in the Soviet show trials and communist POW camps.^2^ Starting in the 1960s, perhaps beginning with *The Manchurian Candidate*, feature films and television series increasingly incorporated pointedly fantastical methods of mind control into their plots, depicting brainwashing through futuristic techniques and technologies.

Many factors drove the trend for incredible, fictional brainwashing imagery, yet significant among them was a transatlantic cultural phenomenon known as ‘spy mania’.^3^ Brainwashing appears in a surprisingly large number of spy-themed film and television productions, many of them created by Anglo-American production teams for English-speaking audiences on both sides of the Atlantic (Burton, 2013; Chapman, 2002). While James Bond himself was never brainwashed on film, he does succumb in Ian Fleming’s 1965 novel, *The Man with a Golden Gun*. In the movie adaptation of *On Her Majesty’s Secret Service* (1969), Bond foils a plot involving women hypnotized to become assassins. Mind control features more frequently in films that followed the Bond formula, such as *The Ipcress File* (1965) and *Our Man Flint* (1966), as well as in espionage-themed TV series like Patrick McGoohan’s cult classic, *The Prisoner* (1967–8); *The Avengers* (1962–9); *The Saint* (1962–9); *Callan* (1967–72); *Man in a Suitcase* (1967–8); *Mission: Impossible* (1966–73); and *The Man from U.N.C.L.E*. (1964–8). That ‘brainwashing’ was perfect for spectacular spy fare was perhaps too obvious – it was lampooned in *Carry On Spying* (1964), *Casino Royale* (1967) and the television show *Get Smart* (1965–70). However, brainwashing was not only for spies, as the plot device was featured in episodes of *Star Trek* (‘Dagger of the Mind’, 1966); *Batman* (‘Fine Finny Friends’, 1966); *The Invaders* (‘The Experiment’, 1967); and *Doctor Who* (‘The Krotons, Part 2’, 1969).

Why did brainwashing feature so frequently in espionage fiction? The literary scholar Timothy Melley has suggested that brainwashing narratives became part and parcel with cold war fantasies of covert operations because both explored the idea, which was fundamental to cold war politics, that in order for democratic order to be preserved, some facts about the state had to be protected from public view. In fiction, cold war spies emerged as the recurrent targets of brainwashing because of what they knew, and how significant it was for their governments that only they knew it (Melley, 2012). In the practical terms of production, however, spy mania was film and television studios’ response to postwar audiences’ diversifying tastes. Cinema audiences had been in decline in both the USA and Great Britain since the Second World War, a trend exacerbated by the flight of middle-class families to the suburbs, and the attendant rise of leisure activities like golf and camping. The greatest threat to cinema-going audiences, of course, was the growing popularity of commercial television. In response, picture houses and film production firms experimented with new wide-screen formats like Cinemascope and Cinerama, and new genres such as musicals and historical epics (Belton, 2013; Hanson, 2007: 87–103). Yet even these innovations were challenged by a fracturing of film audiences by taste, encouraged by the growing variety of hobbies and commercial goods. Emergent subgroups clamoured for their own genres of cinematic entertainment. The surprising box-office success of the first James Bond film, *Dr No* (1962), provided a template for unifying and expanding audiences (Chapman, 2007: 50–5). Many film studios and television broadcasters sought to copy it, often in prescribed fashion. Brainwashing became an accessible and easily reproducible part of the espionage genre.

Many of the visuals and aesthetics that we now associate with cold war mind control date from this explosion of brainwashing imagery within the mainstream. In films and television shows, a victim of brainwashing could be felled through psychoactive drugs, electroconvulsive treatment, sonic rays, or by a colander-like helmet wired to a machine, to name just a few examples. Even hypnosis was given a new gloss. Decades earlier, *The Cabinet of Dr Caligari* (1920) and *Dr Mabuse the Gambler* (1922) had depicted hypnosis on film through close-ups of a hypnotist’s eyes and puppet-master-like hands, thus capturing on film the tradition of identifying hypnotic control with a charismatic individual (Andriopoulos, 2008; Heffernan, 2002). In 1960s portrayals, a victim could be hypnotized by the decidedly uncharismatic efforts of white-coated scientists, or even nameless henchmen, operating a machine, an instrument, or a laboratory-like environment. This was the case in *The Ipcress File*’s brainwashing sequence, as we will see.

Perhaps the formulaic nature of these television shows and film franchises explains why such attention was shown to the mechanisms of brainwashing. By showing the processes, devices and personnel behind a scheme, the narrative could foreshadow how a hero would resist them and restore order. From this perspective, Patrick McGoohan’s 17-episode series *The Prisoner* (1967–8) can be described as the story of a secret agent who is subjected to a sequence of diverse, elaborate methods of brainwashing, and every time finds a way to reassert his integrity as an individual. Interestingly, certain episodes of *The Prisoner* suggested that television itself could be an instrument of surreptitious influence (‘The General’, 1967), or a metaphor for replaying and intervening in memories (‘A, B, and C’, 1967; ‘Living in Harmony’, 1967).

The film scholar Alan Burton has suggested that the diversifying imagery of brainwashing in this period was due to advances in scientific knowledge about what made brains susceptible to coercion, such as the era’s neuropsychological research on sensory deprivation (Burton, 2013). It is true that by the late 1950s, American and Canadian neuroscientists were studying the effects of sensory deprivation as a means to understanding Soviet interrogation methods. Yet the noticeable dearth of fictional portrayals of sensory deprivation indicates why Burton’s explanation may be incomplete. An exception proves the rule: in 1963 a British film *The Mind Benders* (1964) attempted a scientifically realistic portrayal of brainwashing, centring on the psychological effects of sensory deprivation. The film stars Dirk Bogarde as an Oxford physiologist who submits to hours of sensory deprivation in a flotation tank, to show that it can render a person highly suggestible. An official from Military Intelligence intensifies the experiment by persuading the physiologist, at his moment of greatest emotional vulnerability, that his beloved wife Oonagh (Mary Ure) is a debauched ‘tart’. Based on a script by James Kennaway, the film merged elements of British New Wave drama with insights from contemporary scientific research on sensory deprivation. However, the film was unsuccessful with audiences and critics. Contributing to its poor box-office receipts was its certification for adult audiences only, which the British Board of Film Censors awarded because of the film’s serious subject matter – not brainwashing per se, but natural childbirth, marital infidelity and psychological anguish (Aldgate, 1997). Dearden may have pushed the film’s social realism too far, as British audiences’ appetite for ‘kitchen sink’ drama was already waning in 1963 (Nowell-Smith, 2008: 136–8). We might also surmise that sensory deprivation chambers – whether understood to be associated with Soviet interrogation methods or not – were visually uninteresting in a period that offered ever more exciting cinematic entertainments.^4^

All told, in the 1960s the ‘real’ story behind communist brainwashing rarely featured in mainstream movies and television serials, while incredible, futuristic visions of mind control proliferated. With hindsight, it would be simplistic to conclude that this merely reflected audiences’ perverse disregard for scientific opinion. Structural changes within mass media, such as the advent of commercial television and the postwar decline in anglophone film audiences, encouraged film and television producers to innovate new formulas for attracting audiences, such as shooting films in wide-screen formats and revitalizing genres like the spy thriller. *The Ipcress File* stands out among 1960s films and television series not only for how it originated a new approach to depicting brainwashing on film, but for how its approach drew so deliberately on contemporary practices in neuroscience, art and cinematic spectacle.

## Making *The Ipcress File*

*The Ipcress File* is best known for offering a sardonic, unsentimental take on cold war-era British espionage. Released in the UK in March 1965 and a few months later in the USA, it noticeably countered the elitism and patriotism of the James Bond films (Murphy, 1992: 221–2; Shaw, 2001: 61). The film, based on the 1962 novel by Len Deighton, introduced cinema audiences to Harry Palmer (Michael Caine), an insolent, low-level spy who becomes a pawn in a plot to brainwash British scientists (Deighton, 1962). Director Sidney J. Furie’s gritty yet mannered aesthetic for depicting British espionage, and Michael Caine’s cheeky characterization of Agent Palmer, won over audiences in Britain and America and received generally positive reviews. *The Ipcress File*’s 50th anniversary was recently fêted by the British Film Institute with a ‘moviegraphic’ describing its production and significance (Milward-Oliver, 2015a).

*The Ipcress File*’s depiction of brainwashing received less enthusiasm from critics, past and present, despite its originality. In a sequence that comes late in the film, the protagonist Harry Palmer is assaulted with pulsating abstractions of projected light and electronic music, within a cell-like ‘programming box’. That this is meant to be an innovative new method of brainwashing is conveyed in an exchange between the movie’s villain Grantby (Frank Gatliff) and his assistant. The assistant, a man in a white lab. coat who controls the programming box, notes: ‘The Gestapo and the MVD used to beat a man for months to get him to this stage.’ Grantby concurs: ‘That’s old-fashioned and crude. So slow.’ Nonetheless, contemporary reviewers criticized the sequence in terms that aligned it with the hokum of cinematic attractions. John Coleman of the *New Statesman* compared the film’s brainwashing scene with a ‘sadistic circorama’, referring to the 1950s trend for cinemas to show films on a large, curved screen for a more immersive experience. Gordon Gow of the British magazine *Films and Filming* wrote that ‘the only major fault I’d find with *The Ipcress File* is its protraction of an under-motivated brainwashing sequence, presumably dreamed up by somebody who has had the numbing experience of sitting too close to Cinerama’ (Coleman, 1965; Gow, 1965). Meanwhile the reviewer for *The Spectator*, Isabel Quigley, took the film’s director, Sidney J. Furie, to task:

Where I think he [Furie] fails is in being too explicit about the techniques of brainwashing, in making us watch the disintegration of personality through what I thought at first was electric shocks and someone else assured me was merely optical illusions. Which of us is right I still don’t know, but I know Mr. Furie was wrong to arouse such emotional and aesthetic confusion. (Quigley, 1965: 365)

These responses, especially Quigley’s ‘emotional and aesthetic confusion’, suggest that the cinematic imagery of brainwashing that we may now take for granted had, in the mid-1960s, not yet resolved itself into a recognizable visual rhetoric. It was a gimmick, an obvious spectacle – and one that the film’s director, Sidney Furie, had deep reservations about using.

In this section, I will briefly describe the plot of *The Ipcress File* film and detail its depiction of brainwashing. I explain how the sequence came to differ from its source material, Len Deighton’s novel, even as the film’s production team sought to do justice to it. I then provide an account of what likely inspired the brainwashing sequence’s specific innovations, based on original research undertaken by myself and Deighton’s biographer, Edward Milward-Oliver.^5^ This investigation behind the scenes indicates how even spectacular brainwashing imagery could have a close relationship with contemporary scientific practices.

### The brainwashing sequence

In *The Ipcress File* movie, Agent Harry Palmer is a working-class Londoner serving in British Military Intelligence. When he is assigned to investigate a mysterious ‘brain drain’ of top British scientists,^6^ Palmer learns their disappearances may relate to his discovery of an audio tape marked ‘IPCRESS’, which plays a strange, distorted sound. ‘Brain drain’ is revealed as brainwashing when Palmer learns that IPCRESS is an acronym for ‘Induction of Psycho-neuroses by Conditioned Reflex under strESS’. Yet before Palmer can confirm that scientists are being brainwashed, he is kidnapped and subjected to the IPCRESS process.

Harry Palmer’s brainwashing takes place within the ‘programming box’, a man-sized cube surrounded by film projectors and connected to scientific equipment, including an electroencephalogram plotter and oscilloscope. Palmer, who has been kept in solitary confinement with little food or warmth, is strapped into a wheelchair and pushed inside the box. Once Palmer is inside, the box is hoisted above the ground and the projectors direct abstract images through the walls of the box. The IPCRESS noise drones in time with the flashing lights. As Palmer struggles to resist, the villain Grantby hypnotically intones, ‘Relax, relax…listen to my voice, nothing but my voice. You will forget the IPCRESS noise, you will forget all about the IPCRESS file, you will forget your name.’ Palmer tries to distract himself from the sensory overload by yelling his name aloud and gripping a bent metal nail in his fist, causing his palm to bleed. Grantby responds by intensifying the treatment, telling his assistant, ‘I want to fit the rhythm of the sound and vision to the pattern of his brainwaves. I think it will make for a much deeper response.’ As the treatment starts to take effect, the EEG equipment registers Palmer’s brainwaves. Palmer drops the nail from his hand and slumps. The sequence ends when Palmer summons his energy, overpowers his guards and escapes. Later in the film, Palmer returns to the warehouse where his programming took place and overcomes his conditioning by banging his hand painfully against one of the film projectors, a gesture reminiscent of the self-imposed stigmata that helped Palmer initially resist the IPCRESS process.

Len Deighton and Sidney Furie both credit the film’s producer, Harry Saltzman, with *The Ipcress File* film’s take on the mechanisms of mind control. Deighton, who had no control over how his novel was adapted to film, described the brainwashing sequence as ‘flashy’ and compared it, somewhat derisively, with the aesthetic of James Bond (Deighton, 1994). Furie has voiced his own unease, saying ‘The brainwashing machine – it still bothers me. It still embarrasses me’ (Furie and Hunt, 2006). Furie had wished the sequence would follow Deighton’s novel more closely, which meant portraying brainwashing as the stress-inducing techniques used by actual communist brainwashers. Deighton’s own understanding of the mechanisms of brainwashing was strongly influenced by reading a popular scientific monograph by William Sargant, a British psychiatrist who argued that brainwashing was a real phenomenon akin to combat stress (Deighton, personal communication with the author; Sargant, 1957). Yet when it came to the film’s brainwashing sequence, Saltzman’s vision held sway over Deighton and Furie’s. As Furie has explained, ‘It was a long bitter fight. I won most of it but I lost the battle on the final brainwashing scene. Instead of the big gimmicky set and James Bond effects, I wanted a little room and no gimmicks’ (Kremer, 2015: 87).

### From Bond to *Ipcress*

Harry Saltzman, who co-produced the first nine Bond films with Albert Broccoli, purchased the film rights to *The Ipcress File* as Deighton’s novel became a best-seller. Saltzman was hoping to produce a franchise that could capitalize on the popularity of spy films without his having to share profits or creative control with Broccoli. Deighton recalls that Saltzman initially planned to make *The Ipcress File* as a realist, kitchen-sink drama like *Look Back in Anger* (1958) and *Saturday Night and Sunday Morning* (1960), which Saltzman had produced while part of Woodfall Film Productions. But with the worldwide success of the early Bond films, Saltzman sought a spectacular, bigger-budgeted movie that would thrill audiences like the Bond series (Deighton, 1994; Kremer, 2015).

To some extent, Deighton’s novel lent itself to the Bond style. Edward Milward-Oliver has recently uncovered how early plans for the film followed Deighton’s plot closely, and included several scenes that would be filmed overseas in such exotic locations as Beirut and Cape Canaveral (Milward-Oliver, 2015b).^7^ However, these plans abruptly changed just weeks before the start of principal photography, when Columbia Pictures dropped the film from its production slate. Saltzman immediately drew on his relationship with the Rank Organisation, which together with Universal Pictures agreed to provide distribution guarantees sufficient for him to secure a loan from Bank of America to cover a reduced production budget. The production’s straitened financial circumstances dictated that filming be limited to London. A holdover from the earlier, grander production plans was the ‘Albanian prison’ and brainwashing set, which had already been constructed at Saltzman’s insistence at Pinewood Studios (Chapman, 2014; Milward-Oliver, 2015a).

At the centre of the brainwashing set was the ‘programming box’ designed by Ken Adam, who previously had devised iconic sets for *Dr No* and *Goldfinger*, as well as Stanley Kubrick’s *Dr Strangelove or: How I Learned to Stop Worrying and Love the Bomb* (1964). To make *The Ipcress File*, Saltzman brought in much of the Bond series’ production crew: Adam, film editor Peter Hunt, sound editor Norman Wanstall and composer John Barry. To direct, Saltzman hired Sidney J. Furie, a young Canadian whom – Furie’s biographer suggests – Saltzman expected to be deferential (Kremer, 2015: 79). But Sidney Furie was confident in his own abilities and insisted on taking full directorial control. Working with cinematographer Otto Heller, Furie created a bravura visual style, often shooting from behind objects with actors sometimes obscured or out of focus, as if the camera was itself spying on the scene. After nine weeks of shooting in and around London, in mid-November 1964 the production moved to Pinewood Studios and the brainwashing set that awaited them.

### The programming box

Furie and Deighton have said that the overarching concept for the brainwashing sequence came from Saltzman, and that he was inspired by an article in *LIFE Magazine* (Deighton, 1994; Furie and Hunt, 2006). The most probable source was *LIFE*’s September 1962 profile of the ‘Knowledge Box’, a contained space of 12 by 12 feet [3.6 by 3.6 m] created by Ken Isaacs, an instructor in design at the Illinois Institute of Technology in Chicago.^8^ Isaacs presented his Knowledge Box as a prototype for a new mode of multimedia education. A student would enter the box and be fully surrounded by images and text, projected from the 24 slide projectors that peeked into the box from all six sides. The immersive media environment was intended to play upon the mind’s ability to learn through sheer exposure to information. As *LIFE Magazine* explained, ‘It is a machine of visual impact that could depict, for example, a history of the Civil War in a single session, or just as easily give a waiting astronaut a lesson in celestial navigation’ (Welch, 1962: 109).

Saltzman would not have been alone in perceiving Isaacs’s Knowledge Box as a device for mind control. A *Chicago Tribune* reporter compared it with ‘the devilish equipment of a Red-world dungeon devoted to the insidious science of brainwashing’ (Gowran, 1962: 8). Isaacs’s own rationale, which evoked subconscious perception, suggests that Saltzman may have associated the box with ‘subliminal influence’. Subliminal influence had been largely debunked in the late 1950s when James Vicary publicly tested the technology on cinema audiences and failed to prove its effectiveness. Yet public fascination with the possibility of subliminal influence did not disappear, and film-makers experimented with trying to reference the idea of subliminal influence – in liminal ways – to take advantage of the public’s interest (Acland, 2012). In 1962 Harry Saltzman himself helped to organize an experiment in London to test whether subliminal messages in film might enhance cinema audiences’ emotional response, a short-lived cinema trend known as ‘psychorama’ (Heffernan, 2002; *Sight and Sound*, 1962). Saltzman concluded that subliminal messaging would not increase box-office revenues, but his brief interest in psychorama coincides chronologically with the *LIFE* profile of Ken Isaacs’ Knowledge Box, and with his interest in optioning *The Ipcress File* novel.

### Censoring the *IPCRESS* noise

Despite his insistence on the programming box, Saltzman’s vision of the brainwashing sequence initially followed Deighton’s novel in suggesting that the IPCRESS process was physically brutal and that the ‘IPCRESS noise’ was the sound of humans screaming. Yet on this issue Saltzman had to yield to a higher authority, the British Board of Film Censors (BBFC). In September 1964, days before filming began, Saltzman followed standard procedure for British film-makers by submitting the *Ipcress* script to the BBFC for consultation on the film’s likely age certification. Reading an early draft written by Bill Canaway and Johanna Harwood, the censors were concerned that, as the script suggested, Harry Palmer would be beaten ‘methodically’ by his captors and subjected to an ‘IPCRESS noise’ consisting of ‘meaningless and terrible howls’ – the sounds of humans being tortured. One BBFC examiner wrote, ‘Although the story could have been “U” [suitable for all audiences], I doubt whether it will be. There is too much beating and banging about and the stuff in the conditioning cell with the howling tapes would probably be too frightening’ (Examiner’s Report [*The Ipcress File*], 1964). Secretary John Trevelyan wrote to Saltzman confidentially to say that care would be needed even to receive an A rating, allowing children to view the film when accompanied by adults. Referring to the ‘programming sequences’ in the script, Trevelyan writes, ‘Care should be taken with these scenes in which Palmer is treated with brutality. We would not want anything that was prolonged or really vicious. As far as possible the brutality should be suggested rather than shown’ (Trevelyan, 1964).^9^

*The Ipcress File* production team followed Trevelyan’s advice and received the coveted A certification, but this required a new approach to the ‘IPCRESS noise’. Rather than recording natural screams, the film’s sound editor Norman Wanstall asked the BBC Radiophonics Workshop to create the IPCRESS noise with their electronic equipment (Wanstall, personal communication with the author). The Radiophonics Workshop had earlier helped Wanstall create the sound effects for the laser in *Goldfinger* – though such collaborations had to be done after hours and without a formal contract. In composing the IPCRESS noise, Brian Hodgson, a member of the Radiophonics Workshop, viewed an early scene in which the IPCRESS noise is played from a reel-to-reel tape recorder, and then composed a cyclical, rhythmic noise to fit.

### Finding ‘the rhythm of brainwaves’

As Agent Palmer struggles to resist the sensory overload of the programming box, the villain Grantby instructs his assistant: ‘I want to fit the rhythm of the sound and vision to the pattern of his brainwaves.’ This reference to ‘brainwaves’ was a nod to the cutting edge of neuroscience. In the years after the Second World War, neurologists had made strides in encephalography (EEG), the recording of ‘brainwaves’ to investigate the brain’s internal mechanisms. The best-known of these investigations, to contemporary Britons as well as historians of science, were the EEG studies of W. Grey Walter, a neurophysiologist at Burden Neurological Institute in Bristol. In his popular 1953 book, *The Living Brain*, Walter described experiments that he performed with his assistant Harold Shipton in which they used an electronic stroboscopic light with adjustable flicker rate – a technology developed during the Second World War – as a visual stimulus (Walter, 1953). Walter and Shipton recorded the subjects’ brainwaves in response to the flickering light, but also found that subjects began to hallucinate as they watched the rapid flashes, usually by ‘seeing’ colourful figures in motion. This phenomenon led Walter to posit that the brain continually scanned its environment like the raster of a television, a hypothesis that Walter tested by constructing cybernetic robots, his famous ‘tortoises’. Walter’s book became a scientific best-seller in Britain and abroad, in part because of its association with cybernetics and Walter’s own self-promotion (Hayward, 2001).

It is possible that Saltzman or his scriptwriters knew about Walter’s book, which had been reissued by Penguin in 1961. Some of the EEG experiments described in *The Living Brain* were real-life antecedents to the fictional Grantby’s plans ‘to fit the rhythm of the sound and vision to the pattern of his brainwaves’. For these experiments, Shipton created a system of feedback in which the light was set to strobe in synchrony with the frequency of the subject’s alpha rhythms (Walter, 1953: 99). However, when it came time to film *The Ipcress File*’s brainwashing sequence, Saltzman reached out to a different team of researchers for advice.^10^ Saltzman contacted Dr Albert Mason, then a famous anaesthesiologist and medical hypnotist based in London, asking Mason to consult on the film [Mason, personal communication with the author]. At the time, Mason was working with neurophysiologist Martin Halliday on EEG experiments to test whether hypnosis attenuated the transmission of pain signals to the cortex, a hypothesis first suggested by the neurologist George Dawson, a colleague of Grey Walter’s in Britain’s tight-knit community of cyberneticists (Halliday and Mason, 1964; Husbands and Holland, 2008). Mason and Halliday’s experiments used an analog computer of Dawson’s invention to average multiple readings of ‘evoked potential’ in the cortex (Dawson, 1954; Rösler, 2005: 101–3). There were few such ‘Dawson’s averagers’ in the UK at the time, and Saltzman asked Mason if theirs might be borrowed for the filming of *The Ipcress File*. Mason and Halliday loaned the machine to Saltzman for filming. Though the calculative circuitry of Dawson’s averager does not appear in the film, its oscilloscope and multiple-channel EEG plotter are clearly visible, signalling to the film’s audience the sophistication of Grantby’s method of brainwashing.

## Brainwashing the cybernetic spectator

*The Ipcress File* depicts brainwashing by combining projected, pulsating images and electronically synthesized music within an enclosed, bunker-like (even cinema-like) space. Within the programming box, Agent Palmer appears as a spectator, one who not only attends to the visions and sounds forcing their way into his mind, but who also scrutinizes the effects this attention has on his psyche. He pierces his palm with a nail, and repeats his own name, focusing his mind on pain and self-identity to steel himself against incursion. Palmer’s brainwashing begins to take hold only when the sensory stimulation is matched to ‘the pattern of his brainwaves’, seemingly bypassing his conscious resistance – though not entirely successfully, as we learn in the film’s denouement. Palmer ultimately prevails not because of a fundamental loyalty to country, compatriots or political ideology, but because of his own, somewhat perverse self-possession. This melodrama of sensory manipulation and individual consciousness invokes a vision of the human mind as inherently dual: as both vulnerable to environmental conditioning and able to intervene in this reprogramming. *The Ipcress File*’s image of mind control is arguably new and distinctive within the evolving imagery of brainwashing; and it is also representative of a broader perspective on mind and media influence that became apparent in the 1960s, an interpretation of human subjectivity that we might term the ‘cybernetic spectator’.

Admittedly, *The Ipcress File*’s brainwashing sequence may appear merely representative of 1960s fantasies of brainwashing while not immediately conveying the era’s new cybernetic perspectives on media spectatorship and human subjectivity. *The Ipcress File* is just one film among many that represents brainwashing methods by showing a screen-within-the-screen (Killen, 2011). Moreover, the ridicule from contemporary film critics, which compared the programming box with a ‘sadistic circorama’ and other immersive entertainments, reminds us that during the early cold war years there were many environments that asked spectators to self-consciously attend to media’s effects on the mind, and these environments did not necessarily reference new popular or scientific interpretations of mental behaviour, cybernetic or otherwise. Nonetheless many of these environments did: art historians have described *The Ipcress File*’s programming box as just one example of ‘cold war modernism’, one that is comparable with psychedelic art (Mellor, 1993: 185–6; Pavitt and Crowley, 2008: 183–7). It is this affinity with the 1960s psychedelic and counter-cultural art that suggests why the brainwashing sequence in *The Ipcress File* indicates a new popular understanding of the relationship between mind control, mental behaviour and media influence.

At first glance, *The Ipcress File* might seem to offer a mainstream critique, or commercial appropriation, of psychedelia. Its 1965 release roughly coincides with when psychedelic exhibitions and personae began to receive mainstream attention. However, claiming the *Ipcress* brainwashing sequence is ‘psychedelic’ risks overstating Harry Saltzman’s prescience, and fails to grapple with the intellectual genealogy shared by psychedelic art and Saltzman’s innovations – a common lineage represented by Ken Isaacs’ Knowledge Box and Grey Walter’s stroboscopic EEG experiments. By examining this genealogy further, I would like to suggest that we can find a transatlantic cultural fascination with sensory environments that were designed to elicit an emotional, subjective response from inhabitants, as well as instructions to spectators on how to properly attend to their surroundings so as to guide their own mental reprogramming. The artists, theorists and audiences who participated in this emerging culture of spectacle and spectatorship often cited popular cybernetic texts, like Norbert Wiener’s *Human Use of Human Beings* (1950) and Grey Walter’s *The Living Brain* (1953), as well as cybernetic terminology like ‘information’ and ‘feedback’, as they articulated their principles and motivations. More intuitively, they subscribed to a vision of mental functioning that went beyond earlier behaviourist or psychoanalytic interpretations by allowing that environmental stimuli and unconscious desires could condition mental behaviour, but insisted that conscious, creative effort could direct this conditioning toward the subject’s own aims.

Psychedelia, though perhaps best known for its promotion of LSD and other hallucinogenic substances, explored how human-built environments of art, music and architecture could alter states of mind and ‘expand consciousness’ (Blauvelt, 2016; Grunenberg and Harris, 2005; Scott, 2006). Whether or not a spectator took LSD – and many did – such installations were spaces of heightened sensory experience, filled with a pastiche of lights, images and sounds produced by stroboscopes, tachistoscopes, film projectors, tape recorders and even computers. Psychedelic artists appropriated these technologies from their more typical uses in warfare, education and entertainment, turning them toward the counter-cultural project of reprogramming the psyche. Psychedelic environments were designed to delight and disorient, to overwhelm rational, conscious thought by overloading the senses. As the historian Fred Turner explains, psychedelic artists ‘turned toward media, and especially music, multiscreen images, and light shows, to shut down the analytical mind, awaken the unconscious, and allow individuals to come together in communities organized around a shared state of awareness’ (Turner, 2015: 260).

Fred Turner has argued that the 1960s psychedelic artists’ emphasis on immersive, multimedia installations drew from earlier intellectual and artistic attempts to influence the social imaginary of spectators. He traces a genealogy of influence from the New Bauhaus designs of Lazlo Moholy-Nagy to the multi-screen, multi-projector films of the late 1950s and 1960s, such as Ray and Charles Eames’ *Glimpses of the USA* (1959); and from there to the psychedelic ‘Movie-Drome’ that Stan VanDerBeek designed and built in upstate New York in 1965. Turner calls these spaces ‘democratic surrounds’ because of their utopian intent. Within the history of ‘democratic surrounds’ that Turner describes, we can locate Ken Isaacs’ Knowledge Box, the source of inspiration for Saltzman’s ‘programming box’ in *The Ipcress File*. Indeed, Isaacs’ biography follows Turner’s account precisely. When he created the Knowledge Box in the early 1960s, Ken Isaacs was an instructor of design at the Illinois Institute of Technology, the home of Moholy-Nagy’s New Bauhaus. Stan VanDerBeek reportedly visited Isaacs’ Knowledge Box in Chicago before creating his iconic theatre of ‘expanded cinema’, the Movie-Drome (Snodgrass, 2016: 373). But most importantly, Isaacs’ Knowledge Box is easily compared with Ray and Charles Eames’ experiments in multi-screen film, and shares similar references to cybernetics and information theory. In the Knowledge Box, as with the Eameses’ films, we find examples of how 1960s democratic surrounds often came with instructions to the audience on how to experience them properly, instructions that frequently drew on cybernetic texts and ideas for justification.

The Knowledge Box was not a singular design for Isaacs, but an elaboration of the Matrix Drum, a structure that Isaacs created while an instructor at Cranbrook Academy of Art in the 1950s. At Cranbrook, Isaacs honed his ‘matrix’ philosophy of design, which was deeply influenced by his reading of Norbert Wiener’s *The Human Use of Human Beings* (Selim, 2014). From Wiener, Isaacs adopted a belief in the levelling, liberating power of ‘information’, later expressing this as ‘The only humane and effective way to break the negative grip of antique culture is with INFORMATION’ (Isaacs, 1970). Indeed for Wiener, the flow of information in systems could bring order, purposiveness and progress, even as such systems evolved beyond any one individual’s or society’s control. Arbitrary hierarchies would be demolished by the natural exchange of information, and new, stable organizations would form. Interpreting Wiener’s writings in psychological terms, Ken Isaacs saw film, images and architecture as potentially rich with information, and a means to challenge the spectator to create her or his own meaningful connections between disconnected facts and ideas. Isaacs hoped that a student who entered the Matrix Drum or Knowledge Box would be alert, not passive, to the flurry of words, images and sounds encircling her, that she would acquiesce to her intellectual transformation by cognitively directing the process (Welch, 1962). A similar principle of spectatorship, also inspired by cybernetic philosophy, informed Ray and Charles Eames’ multi-screen films, *Glimpses of the USA* (1959) and *Think* (1964) (Colomina, 2001; Turner, 2015: 279–81).

The need to offer guidance to the spectators of democratic surrounds continued in the psychedelic counter-culture, as did reference to cybernetic interpretations of mind and media. Individuals who were new to psychedelia’s ‘happenings’ often did not know what to make of these performances, or how best to translate the sensory experience into expanded consciousness. Psychedelia’s spokespersons offered instructions to the uninitiated, imposing their own culture of spectatorship – of how best to experience psychedelia – as a means to ‘free’ individuals from their social conditioning (Shortall, 2014). Michael Hollingshead, the Englishman ex-pat in Timothy Leary’s retinue, recalls staging a happening in New York City’s Greenwich Village in April 1965. He began the event by telling the audience:

Our purpose in being here is to expand our awareness…Tonight we shall be mixing auditory and visual phenomena. The brain is capable of processing all this data. It will see different images moving in a random/planned fashion. Sound tracks, some of which have been cut up, will be heard. Films and light will perform. All you have to do is focus on one point. And then you will see the rest. Diversity will be unity. But do not try to understand. The brain will do all that later. Here you will have 10,000 visions. So sit back and relax. Extend yourself to an aesthetic distance. You may have the opportunity of leaving your body. Leaving your mind. You are going on a voyage. The price of admission is your mind. For if you attempt to analyse and conceptualise you will cheat yourself of the opportunity to see things in a fresh manner. (Hollingshead, 1973: 113)

For Hollingshead, Timothy Leary and many other leaders of the psychedelic counter-culture, the rational mind needed to be transcended and recalibrated. They did not counsel passivity in the face of media, however, but an active search for, and construction of, immersive media environments that could expand consciousness and enable the rational mind to be reprogrammed. Like Isaacs and the Eameses, the psychedelic counter-culturalists drew on popular cybernetic texts, aided by interpreters like Marshall McLuhan, Buckminster Fuller and Gene Youngblood (Turner, 2010). Even Timothy Leary, who promoted taking LSD as the nonpareil instrument of enlightenment, suggested in his 1965 paper ‘Languages: Energy Systems Sent and Received’ that collages of film, images and sounds might be used to communicate and reconstruct one’s transcendent, mind-altering experiences for others (Leary and McGlothlin, 1965).

The ‘cybernetic spectator’ who emerges in these cold war-era multimedia surrounds was perhaps ‘cybernetic’ in ways that would be unrecognizable to Norbert Wiener and other early pioneers of cybernetic science. Counter-cultural interpretations of cybernetics are far removed from the mathematical rigor of Wiener and Claude Shannon’s theories of information, and indeed from the military-industrial complex that fostered their ideas. However, a more direct line can be traced from cybernetic science to the image of mental reprogramming depicted in *The Ipcress File* and psychedelic art, and to the figure of the cybernetic spectator that inhabits both. This line passes through the EEG research of W. Grey Walter, the British neuropsychologist and cybernetician, and in particular his 1953 book *The Living Brain.* As noted above, Walter’s experiments with EEG, described in *The Living Brain*, foreshadowed *The Ipcress File*’s portrayal of hypnotic programming as a matter of matching flashing lights and pulsing sounds to the ‘pattern of brainwaves’. Walter’s book also influenced certain counter-cultural ventures. For instance, the EEG experiments described in *The Living Brain* inspired Brion Gysin, William S. Burroughs and Ian Sommerville to create ‘the dreamachine’, a home-made stroboscope that could elicit hallucinations as reliably, if not as intensely, as LSD (Geiger, 2003). Walter’s book also steered the artist and musician Tony Conrad as he made *The Flicker* (1965–6), an experimental film that used structural properties of film projection to induce psychedelic effects (Canales, 2011; Pickering, 2010). Though a ‘cybernetic spectator’ may not be readily apparent in these offspring of Walter’s EEG research, it nevertheless surfaces when considering how Walter, in *The Living Brain*, interprets the emotional experiences of the human subjects of his experiments, and presents his results in an overarching account of the mental processes of the human brain.

In Walter’s experiments with stroboscope and EEG, subjects were asked to describe their hallucinations as they occurred, including their emotional response, which Walter observed could take the form of fatigue, confusion, fear, disgust, anger, or pleasure. Yet, a subject’s emotions were not without conscious mediation, as the subject could ‘consciously and with effect resist or give way to the emotions or hallucinations engendered by the flicker’, thus heightening a subject’s experience of pleasure or discomfort (Walter, 1953: 68). This was not an idle observation, but rather the key – as Walter describes it – to discovering what the alpha rhythm was tracking as the brain scanned its environment. The alpha rhythm, when correlated in time with a subject’s emotional status, showed that the brain was *learning*, even in the absence of meaningful sensory information:

When the stimulus is first presented, as far as the subject is aware it might imply anything – or be implied by anything; but as flash succeeds flash in monotonous series, the possibility of significance is dismissed. That is, unless the whole situation takes on a peculiar character – of pleasure or discomfort. Then the response may augment and spread and the subject complain that ‘the light makes my head ache’, drawing a reasonable conclusion of causality which experiment shows to be false. (Walter, 1953: 133–4)

From these experiments, Walter concluded that the human mind was fundamentally curious, and conditionable, even when faced with the meaninglessness of strobing light.

In *The Living Brain*, the human subjects of Walter’s EEG experiments come to represent the minds of everyday citizens. The normal, healthy mind was actively and creatively engaged with its surroundings. Meanwhile, the pathological mind was that of the ‘child gazing at the screen of a television receiver’, imbibing information without actively seeking or engaging with it (1953: 186). In a similar vein, Walter criticized ‘totalitarian’ education, as he believed might be found in Russia, which tried to teach the mind not to question, not to learn. This, Walter claimed, would invariably lead individuals to neurotic breakdown (ibid.: 184). Grey Walter’s cybernetic interpretation of the mind thus imagined a spectator who was always open to reprogramming by his or her environment but might nevertheless guide the course of this reprogramming through conscious effort. To resist conditioning was pathological, if not futile, and thus mental freedom lay in actively responding to, and helping to fashion, one’s sensory environment.

## Conclusion

*The Ipcress File*’s elaborate brainwashing sequence was a product of reimagining mind control amid 1960s ‘spy mania’, a transatlantic phenomenon that combined spectacular, cinematic entertainment with the captivations of the cold war national security state. Yet the film’s specific innovations – enclosed spaces, droning rhythms, flashing lights – are more typically construed as part of another transatlantic movement, the 1960s psychedelic counter-culture. This resemblance is instructive, even if accidental. By the late 1960s, psychedelic art and brainwashing imagery converged on the mind’s need for sensory input and its capacity for reprogramming. Both could draw on a cybernetic interpretation of the mind to express how perception and consciousness were dynamically related. Cybernetic philosophy rendered morally ambivalent the scenario of the human spectator, encased in her or his sensorium, faced with resisting or giving way to perception – however, it implied that the spectator could make a conscious choice. The cybernetic spectator thus differed from other visions of human subjectivity that gave less credence to individuals’ power to wilfully reprogramme themselves.

Brainwashing had long raised the question of what made psychological control possible, whether one’s environment was a torture chamber, a totalitarian society, or a culture rife with mass media. The 1960s counter-cultures offered an alternative: the need for individuals to participate in the design and construction of these environments, to guide them with emancipatory intent. *The Ipcress File*, and other 1960s representations of brainwashing, countered that such spectacles could still be coercive – sometimes entertainingly so. This ambivalent imagery, which I have argued was encouraged by transatlantic developments in mass visual media, counter-cultural art and the mind sciences, was an evolutionary step beyond the original meanings of ‘brainwashing’ found in 1950s American popular culture. It has since become a recognizable part of brainwashing’s cultural imaginary.

## Notes

This article was first presented as a paper at ‘Brainwash: History, Cinema and the Psy Professions’, a workshop held in July 2015 at Birkbeck, University of London. The workshop was organized by the Birkbeck Institute for the Moving Image and by The Hidden Persuaders Project. I wish to thank my anonymous reviewers, Edward Milward-Oliver and my colleagues in The Hidden Persuaders Project, Daniel Pick, Sarah Marks, Charlie Williams, Ian Magor and Katie Joice, for their help as I revised this article for publication. I dedicate this article to my mentor and friend Alison Winter (1965–2016).

1 Ron Robin argues that behavioural scientists like Biderman were influenced by scientism and a desire for patronage in constructing their deflationary interpretations of communist brainwashing techniques (Robin, 2001). Nevertheless, reports of communists’ potential methods – not only real ones – led the CIA to support scientific research, such as Project MKULTRA, that was often unethical and sometimes bizarre (Lemov, 2011, 2015).

2 For historical analysis of films depicting the plight of American POWs in the Korean War, see Young (1998).

3 ‘Spy mania’, as I am using the term, was not a mere resurgence of popularity for stories about espionage. Rather, it should be understood more narrowly as a trend or ‘cycle’ in 1960s cinematic and televisual entertainment: spy mania combined long-standing narrative conventions of espionage fiction with patterns of media production and consumption that were distinctive to the 1960s and early 1970s. For an account of similar ‘cycles’ in film history, see Stanfield (2015).

4 Sensory deprivation would not feature memorably in a film or television series until Ken Russell’s *Altered States* (1980).

5 I am indebted to Edward Milward-Oliver for sharing his notes and contacts with me, and encouraging me in my own investigation of how *The Ipcress File* brainwashing sequence was created. All inaccuracies and errors of interpretation are my own.

6 The London *Evening Standard* first coined the phrase ‘brain drain’ in 1963 amid public concern that too many talented British scientists were moving to the United States (Godwin, Gregory and Balmer, 2009).

7 Over a period of 15 months, six writers worked on the *Ipcress* screenplay: Lukas Heller, Lionel Davidson, Ken Hughes, Johanna Harwood, Bill Canaway and James Doran. Early drafts have been lost, but Edward Milward-Oliver discovered in the diaries of Lionel Davidson and the private correspondence of Bill Canaway that initial plans for the film retained the overseas locations described in Deighton’s novel, though the fictional Tokwe Atoll was replaced by the NASA launch complex at Cape Canaveral.

8 The *LIFE Magazine* archive has been digitized and is searchable online through Google Books. My searches for ‘sensory’ ‘programming’ and ‘brainwashing’ suggest that Isaacs’ Knowledge Box is by far the most likely inspiration for Saltzman’s programming box. Moreover, the boxes appear strikingly similar.

9 Saltzman had prior experience negotiating with John Trevelyan and the BBFC examiners about what constituted acceptable violence, due to the Bond films (Trevelyan, 1973: 158).

10 Excerpts from my conversation with Albert Mason are accessible @: http://www.bbk.ac.uk/hiddenpersuaders/blog/albert-mason-medicine-magic-hypnotism/ (accessed 12 February 2017).
